# Biomarkers of Endothelial Damage and Disease Severity in COVID-19 Patients

**DOI:** 10.3390/cimb47060409

**Published:** 2025-05-31

**Authors:** Panagiota Tsiatsiou, Dimitrios Pilalas, Vasiliki Tsaireli, Antonia Lanta, Georgios Meletis, Angeliki Kassomenaki, Evangelia Tza, Lampros Tampakas, Helen Gkeka, Maria Papaioannou, Efthymia Protonotariou, Lemonia Skoura

**Affiliations:** 1 Department of Microbiology, AHEPA University Hospital, School of Medicine, Aristotle University of Thessaloniki, 54636 Thessaloniki, Greece; 2 1st Department of Internal Medicine, AHEPA University Hospital, School of Medicine, Aristotle University of Thessaloniki, 54636 Thessaloniki, Greece; 3 Department of Intensive Care Unit, AHEPA University Hospital, 54636 Thessaloniki, Greece; 4 Division of Hematology, First Department of Internal Medicine, AHEPA University Hospital, School of Medicine, Aristotle University of Thessaloniki, 54636 Thessaloniki, Greece

**Keywords:** P-selectin, COVID-19, endothelial damage, angiogenesis

## Abstract

The severe outbreak of SARS-CoV-2, the etiological agent of COVID-19, has precipitated the development of vaccines and antiviral therapeutics. However, it remains a significant public health concern. This study investigated the association between disease severity, biomarkers of coagulation, and endothelial damage, including P-selectin, thrombomodulin, PAI, von Willebrand antigen (VWF: Ag) and von Willebrand factor ristocetin cofactor (VWF: RCo). A cross-sectional, observational study was conducted in a cohort of 90 adult COVID-19 patients (≥18 years), categorized into three groups: ICU-hospitalized, non-ICU hospitalized, and asymptomatic non-hospitalized (outpatient). In these groups, biomarkers, including PAI-1, TM, and P-selectin, were assessed using enzyme-linked immunosorbent assay (ELISA), and immunological assays for VWF: Ag and VWF: RCo. Across all groups, we observed significantly elevated levels of P-selectin, VWF: Ag, and VWF: RCo. Elevated levels of PAI-1 and TM were observed in ICU patients compared to non-ICU and asymptomatic patients, indicating increased endothelial injury and activation. Furthermore, COVID-19 mutations significantly affect the P-selectin biomarker. This finding supports the hypothesis that P-selectin is a more reliable biomarker for assessing the severity of the disease than other endothelial damage and coagulation markers, especially in heterogeneous clinical presentations. Our study also highlights the requirement of comprehensive examination for its broader implications in viral strains, infection severity, and genetic variants.

## 1. Introduction

The global outbreak of severe acute respiratory syndrome coronavirus 2 (SARS-CoV-2), the pandemic’s underlying cause of the coronavirus disease 2019 (COVID-19), has caused a traumatic loss of life and disrupted numerous aspects of social life. The development of vaccines and antiviral drugs has reduced the disease burden. In May 2023, the World Health Organization (WHO) announced that COVID-19 was no longer a public health emergency of international concern globally.

However, COVID-19 is still a constant challenge. SARS-CoV-2 is distributed in communities, and new variants with the potential to escape the immune system caused by the vaccine have appeared. All vulnerable patients are still at risk of serious infections. Post-acute sequelae of SARS-CoV-2 infection remain a significant public health concern, affecting 9.6% of infected patients [1].

The current understanding of COVID-19 pathogenesis suggests alveolar epithelial injury can induce endothelial activation and apoptosis through various stimuli, including hypoxia, damage-associated molecular patterns, cytokines, and chemokines. Exposure to the extracellular matrix induces fibrin deposition through intrinsic and extrinsic coagulation pathways. Activated platelets bind to the extracellular matrix and stimulate neutrophils and monocytes to release neutrophil extracellular traps (NETs). Interplays between activated platelets, neutrophils, monocytes, and NETs facilitate coagulation and formation of fibrin thrombi via an immune-mediated mechanism known as immunothrombosis. Fibrinolysis may also be impaired [2,3,4].

Additionally, endothelial injury promotes a prothrombotic state, increasing the risk of thromboembolic events, microthrombosis, and macrothrombosis. Comprehensive research has not yet identified reliable biomarkers for stratifying the severity of the disease. Complex clinical presentations are difficult to diagnose and manage because there are no established biomarkers. This study assessed endothelial dysfunction and platelet activation biomarkers, including PAI, thrombomodulin, von Willebrand antigen (VWF: Ag), von Willebrand factor ristocetin cofactor (VWF: RCo), and P-selectin, to find a reliable biomarker to predict disease severity in COVID-19 patients.

Plasminogen activator inhibitor-1 (PAI-1) is an inhibitor of the plasminogen activator (tPA) and urokinase-type plasminogen activator (uPA). PAI-1 limits the production of plasmin, which is the main enzyme involved in fibrinolysis, and degrades fibrin [5]. In COVID-19, damage to alveolar type II cells leads to lower surfactant levels, higher p53 expression, and higher PAI-1 levels, promoting thrombosis and reducing fibrinolysis [6].

Thrombomodulin (TM) is a 557-amino-acid-containing transmembrane protein. The dysregulation of this gene, which has been associated with COVID-19, has also been implicated in endothelial damage, anticoagulant function loss, and a prothrombotic phenotype, which may be responsible for the thrombosis that occurs in patients with COVID-19 [7,8].

The von Willebrand factor (VWF) plays a significant role in inflammatory responses since it binds to neutrophil extracellular traps (NETs) to enable the recruitment of leukocytes to vascular damage or inflammation sites. VWF also binds to platelets, particularly in the context of thrombus formation. Plasma levels of (VWF: Ag) and (VWF: RCo) are significantly elevated in patients with COVID-19, which correlates with poor prognosis, increased severity, and higher mortality rates [9,10].

P-selectin is also a marker of endothelial damage and platelet activation in patients with COVID-19. Elevated expression of P-selectin in COVID-19 patients enhances neutrophil adhesion and platelet aggregation, which may contribute to coagulopathy. In addition, numerous studies have shown that the role of P-selectin in long-term COVID-19 binding leads to a correlation between P-selectin and the persistence of symptoms [11,12].

We conducted an exploratory observational study to evaluate the association of selected biomarkers implicated in hemostasis and endothelial damage with the severity of COVID-19 presentation. Our analysis identified P-selectin as a promising biomarker and has also shown improved diagnostic and prognostic value compared to other platelet activation and endothelial damage biomarkers. The finding foresees the future application of P-selectin as an early diagnostic marker for risk stratification, monitoring of disease progression, and facilitating personalized medicine not only in COVID-19 patients but also in other diseases in which endothelial dysfunction plays a critical role.

## 2. Materials and Methods

### 2.1. Study Design and Patient Characteristics

Between 20 July 2021 and 30 December 2022, 90 adult COVID-19 patients (≥18 years) participated in this cross-sectional, observational study at AHEPA University Hospital, a 700-bed tertiary care facility located in Thessaloniki, Greece, which served as one of a referral hospital for COVID-19 patients in Northern Greece during the pandemic. Various virus strains have been identified, but the research material is on Delta and Omicron variants.

The study cohort consisted of 90 individuals diagnosed with COVID-19, divided into three primary groups: 30 patients hospitalized in intensive care units (ICUs), 30 patients admitted to general wards (non-ICU), and 30 asymptomatic individuals who did not require hospitalization (outpatient). The asymptomatic group comprised patients identified through contact tracing following exposure to symptomatic COVID-19 cases and were subsequently confirmed positive for SARS-CoV-2 via precautionary testing conducted at AHEPA Hospital.

To investigate the association between disease severity and selected biomarkers—specifically focusing on intra-infection variability rather than differences between infected and non-infected individuals—all participants included in this study had confirmed exposure to SARS-CoV-2, thereby minimizing potential confounding related to infection status. The three primary groups (ICU, non-ICU, and asymptomatic) were further stratified by year of diagnosis (2021 or 2022), resulting in six subgroups of 15 patients each: asymptomatic 2021, asymptomatic 2022, non-ICU 2021, non-ICU 2022, ICU 2021, and ICU 2022.

The analysis focused on identifying statistically significant differences among these subgroups concerning the P-selectin index. Each participant was included only once in this study to avoid repeated measures or overlapping data, as no individual experienced reinfection during the study period. Based on real-time reverse transcription PCR (RT-PCR) in nasopharyngeal swab samples, COVID-19 was identified.

The AHEPA Hospital Research Ethics Committee (129/19 March 2020) and the Bioethics Committee of Aristotle University both approved this study. The Helsinki Declaration was adhered to in all procedures conducted on patients. Written informed consent was obtained from all patients, including those of relatives or guardians of the ICU patients.

In the study enrollment information, the patient’s age, sex, symptoms, medical history, and laboratory findings were reported. Patients younger than 18 years with thrombophilia, liver disease, renal failure, or a critical illness related to another diagnosis other than COVID-19 that affects the thrombotic status were not included in this study.

### 2.2. Blood-Sampling Procedures

Venous blood was obtained 72 h after admission to the ward and intensive care unit. As indicated by the test, blood samples were collected into plastic vacutainer tubes with 0.129 M (3.8%) trisodium citrate (catalog number 363095; Becton Dickinson, Dublin, Ireland) for plasma samples in a 9:1 blood-to-anticoagulant ratio.

The test samples were centrifuged twice at 2500× *g* for 15 min at 18–25 °C to obtain platelet-poor plasma. For procedures that require serum samples, venous blood samples are also collected in vacutainer tubes coated with silicon and micronized silica particles to facilitate clotting (catalog number 367837; Becton-Dickinson, Dublin, Ireland). Plasma was obtained, divided into 0.5 mL portions, and stored at −80 °C until use. The samples were then frozen until further use. The occurrence of freeze–thaw cycles was prevented.

### 2.3. Laboratory Procedures

Standard coagulation assays, such as prothrombin time (PT), activated partial thromboplastin time (aPTT), fibrinogen, D-dimer, and coagulation factor VIII, as well as for endothelial biomarkers von Willebrand antigen (VWF: Ag) and von Willebrand factor ristocetin cofactor (VWF: RCo), were conducted using the ACL TOP 50 series instrument (Instrumentation Laboratory, Bedford, MA, USA) with the appropriate reagents and controls as defined by the laboratory protocol.

P-selectin, thrombomodulin, and plasminogen activator inhibitor (PAI) are laboratory markers of endothelial dysfunction and platelet activation. The antigen levels of P-selectin and thrombomodulin/BDCA-3 were measured using an enzyme-linked immunosorbent assay (ELISA) according to the manufacturer’s instructions (Novus Biologicals, Centennial, CO, USA). The antigen levels of PAI were also assessed using the RnD Systems Human Total Serpin E1/PAI-1 immunoassay (ELISA) (RnD, Minneapolis, MN, USA): thrombomodulin/BDCA-3 (sensitivity: 27 pg/mL; range: 2866–5318 pg/mL; mean value: 4032 pg/mL; SD: 637 pg/mL; cross-reactivity < 0.5%), P-selectin (sensitivity: 0.121 ng/mL; range: 43.5–119 ng/mL; mean value: 82.6 ng/mL; SD: 20.0 ng/mL; cross-reactivity < 0.5%), PAI (sensitivity: 0.142 ng/mL; range: 0.3–20 ng/mL; mean value: 5.10 ng/mL; SD: 3.74 ng/mL; cross-reactivity < 0.5%). All samples were assayed in duplicate. The biomarkers are presented as standard reference ranges and ratios.

### 2.4. Statistical Analysis

This study employed a parametric independent sample *t*-test or non-parametric Mann–Whitney U test to evaluate the mean differences among patient groups. Also, a Spearman’s rank correlation was used to assess the relationship between vaccination and disease severity and vaccination status and P-selectin levels. Ordinal logistic regression was used to analyze P-selectin index differences among the six subgroups based on 2021 and 2022, considering varied COVID-19 mutations. Data analysis was conducted using the IBM SPSS Statistics software version 29.

Ordinal logistic regression was used to identify the independent variables affecting patient severity. Collinearity diagnostics estimates the variance inflation factor (VIF) and identifies variables that may cause collinearity issues in the model. PT, INR, von Willebrand antigen (VWF: Ag), and von Willebrand factor ristocetin cofactor (VWF: RCo) were excluded from the model due to their high VIF values (see Supplemental Data Table S5).

## 3. Results

Our study, meticulously conducted among 90 individuals, categorized 51 males (56.7%) and 39 females (43.3%) into three groups based on disease severity. Chronological grouping was also conducted, spanning examinations from 2021 to 2022. Patient characteristics, including age and sex, were consistent across the groups (Table 1). Also, group comparisons of the P-selectin index were performed both across the three main severity groups and across the six subgroups combining severity and year of diagnosis. History of infection, comorbidities, and vaccination were reported (see Supplemental Data Tables S1–S3). Given the large number of unvaccinated patients and those vaccinated with one dose, in our study, the association between vaccination status and disease severity by Spearman’s rank correlation showed no significant relationship (ρ = 0.068, *p* = 0.526), indicating that vaccination status alone does not distinguish between clinical severity groups in this cohort. Finally, no significant correlation was found between vaccination status and P-selectin levels (Spearman’s ρ = 0.069, *p* = 0.529), implying that vaccination did not have a direct measurable effect on this biomarker of endothelial activation in our study population.

The mean and SD of hematological parameters of complete blood count in the total number of patients, asymptomatic non-hospitalized (outpatient), non-ICU, and ICU are shown in Table 2. We found a statistically significant difference in the white blood cell count, neutrophil absolute count, and neutrophil-to-lymphocyte ratio (NLR) (normal values 0.78–3.53) among the three clinical groups, with *p*-values less than 0.05, providing significant insights into disease severity.

Our comprehensive data analysis revealed further insights. The coagulation parameters PT, aPPT, INR, and fibrinogen did not reveal statistically significant differences across all pairs of patient groups, while the D-dimer, coagulation factor VIII, von Willebrand antigen (VWF: Ag), and von Willebrand factor ristocetin cofactor (VWF: RCo) showed a statistically significant difference between the outpatient and non-intensive care unit (ICU), outpatient and ICU, and non-ICU and ICU subgroups (*p* < 0.001) as obtained from the Mann–Whitney U test. Mean values for PT, INR, aPTT, fibrinogen, D-dimer, FVIII, VWF: Ag, VWF: RCo, PAI, and thrombomodulin for each patient group are shown in Supplemental Data Tables S4 and S5.

Also, regarding plasminogen activator inhibitor-1 (PAI) and the thrombomodulin index, according to the non-parametric Mann–Whitney U test, there was a statistically significant difference between the ICU and the other two subgroups (outpatient and non-ICU), but there was no statistically significant difference between the outpatient and non-ICU subgroups. Statistically significant differences are indicated by *p*-values less than 0.05.

Ordinal logistic regression with seven biomarkers (PAI, thrombomodulin, P-selectin, aPTT, fibrinogen, D-dimer, and FVIII) predicting patient severity yielded a strong model fit (*p* < 0.001). The goodness-of-fit test supported model adequacy (*p* = 1), indicating data alignment with the predictions. The pseudo-R-Square (McFadden) was 0.621, reflecting a substantial improvement of 62.1% over the null model. Significant coefficients were observed for P-selectin (*p* < 0.001), D-dimer (*p* = 0.004), FVIII (*p* = 0.003), and PAI (*p* = 0.009), with estimates of 0.670, 0.002, 0.017, and 0.291, respectively, indicating higher index values associated with increased severity likelihood. The test of parallel lines (*p* = 0.254) validated a consistent relationship between predictor variables and the outcome, ensuring interpretation without violating the proportional odds assumption (see Supplemental Data Table S6).

The ordinal logistic regression model, using only the four significant variables (P-selectin, D-dimer, FVIII, and PAI), displayed a robust fit (*p* < 0.001) with further validation through a *p*-value of 1. Sex was not included because was not a significant predictor *p* = 0.669. The pseudo-R-Square (McFadden = 0.561) indicated a substantial 56.1% improvement over the null model. All variables showed significance (*p* < 0.05), with estimates of 0.624, 0.002, 0.017, and 0.343 for P-selectin, D-dimer, FVIII, and PAI, respectively (see Supplemental Data Table S7). The test of parallel lines yielded a non-significant *p*-value of 0.386, suggesting no violation of the proportional odds assumption.

Considering the six subgroups of patients (samples of 15 individuals) and the measurement dates of the biomarker (2021 and 2022), we found that differences were considered statistically significant at *p*< 0.05. The mean, SD, and median of the P-selectin biomarkers for the six subgroups of patients (asymptomatic 2021 (outpatient 2021), asymptomatic 2022 (outpatient 2022), non-ICU 2021, non-ICU 2022, ICU 2021, and ICU 2022) are shown in Table 3.

Our analysis of the P-selectin index across six patient subgroups, spanning the years 2021 and 2022, revealed potentially significant disparities. Concerning the P-selectin index, the effect of the different COVID-19 strains was recorded, indicating that the mutations impacted significantly the P-selectin index. This finding could have significant implications for understanding the impact of COVID-19 mutations on disease severity.

## 4. Discussion

Our research has unveiled crucial insights through an exploratory analysis of cross-sectional observation data. In our research into COVID-19, we investigated the relationships between endothelial dysfunction, platelet activation, and patient status and presented interesting and informative results.

Our study, which evaluated standard routine analysis and biomarkers of endothelial dysfunction and platelet activation, such as P-selectin, thrombomodulin, PAI, von Willebrand antigen (VWF: Ag), and von Willebrand factor ristocetin cofactor (VWF: RCo), identified a reliable biomarker for predicting disease severity. The coagulation parameters PT, aPPT, INR, and fibrinogen did not reveal statistically significant differences across all pairs of patient groups. D-dimer, coagulation factor VIII, VWF: Ag, and VWF: RCo showed statistically significant differences between subgroups. Regarding PAI and the thrombomodulin biomarkers, there was a statistically significant difference between the ICU and other two subgroups (outpatient and non-ICU). Research on coagulation parameters in COVID-19 patients has yielded varying results. Our study has exhibited no significant differences for PT, PTT, INR, or fibrinogen, making them insufficient as disease severity markers [13,14].

Regarding vaccination, during this study, the limited number of vaccinated individuals, as well as those with only one dose, combined with the presence of highly transmissible variants (Delta and Omicron) and the partial immune escape of the vaccines, led to insufficient population coverage, to the extent that the outcome of the result was not affected. Our research confirms the important role of the booster in reactivating the immune system, highlighting the need to administer and develop vaccines that can address emerging variants.

Moreover, in our study, P-selectin was revealed to be an optimal and stable biomarker of endothelial dysfunction and platelet activity, demonstrating a consistent pattern for predicting disease severity among patients with different clinical characteristics, such as in the Delta and Omicron variants, compared with other coagulation biomarkers. Our findings align with those of previous studies conducted by Bongiovanni et al., Solomon et al., Al-Tamimi et al., Agrati et al., and Campo et al. [12,15,16,17,18]. Several studies have reported that high P-selectin levels are associated with a poor prognosis in patients with COVID-19 [19]. In addition, Vassilliou et al. found that critically ill COVID-19 patients with lethal disease had elevated ICU P-selectin entry levels [20]. Moreover, Garcia et al. reported that soluble P-selectin (sCD62P) was significantly increased in the plasma of patients infected with the Omicron variant compared to healthy controls. However, studies examining P-selectin stability in patients with COVID-19 during the Delta and Omicron waves are scarce [21]. It has been observed that elevated levels of P-selectin are present in the blood of individuals with a range of diseases, such as acute and chronic cardiovascular diseases, hypertension, acute myocardial infarction, deep venous thrombosis (DVT), diabetes mellitus, sickle cell disease, etc. [22,23,24,25]. When assessing the utility of P-selectin as a biomarker for the severity of COVID-19, it is crucial to consider individual patient characteristics, different stages of the disease, and comorbidities. This process ensures that P-selectin level assessments provide critical insights into the severity of the disease, allowing for healthcare professionals to make informed decisions and provide appropriate care to people affected by COVID-19 variants, thus avoiding thrombosis complications. These findings have significant implications for managing and treating COVID-19 patients, particularly those at risk of thrombosis. This study identified elevated levels of white blood cells and absolute white blood count in ICU patients, and the neutrophil/lymphocyte ratio was elevated among groups.

These results are consistent with the literature, which indicates that these increases are often related to lung and systemic cell activity in patients with COVID-19 and are caused by immune responses. This research shows that the pathogenesis of SARS-CoV-2 infection inducing a coagulation path leads to microthrombosis, macrothrombosis, and subsequent fibrin decomposition. Finally, this process increases the level of D-dimer. The literature suggests that high levels of D-dimer are associated with severe disease and increased mortality in COVID-19 patients. Therefore, D-dimer is considered a reliable predictor marker [26,27].

Also, our study demonstrated that the serum levels of factor VIII of VWF: Ag and VWF: RCo are positively correlated with the severity of the disease [28]. According to Zhang et al., the von Willebrand factor demonstrated the strongest association with in-hospital severity mortality in COVID-19 patients [29]. Also, Helms et al., Ladikou et al., Bray et al., and Rostami et al. evaluated the thrombotic risk in COVID-19 patients and found that the levels of WF and VIIIc were significantly elevated in these patients [28,30,31,32]. They are comparable to those seen in ICU patients with severe sepsis. According to our findings, the median VIII, VWF: Ag, and VWF: RCo in ICU patients were similar to those found in critically ill patients with septic shock and severe sepsis and much more significant than healthy controls, according to Hovinga et al. [33]. These findings suggest that the levels of factor VIII, VWF: Ag, and VWF: RCo could be used as biomarkers for predicting disease severity in COVID-19 patients.

Concerning evaluating the fibrinolytic system, in our research, PAI levels were higher in ICU patients than in non-ICU and asymptomatic groups (outpatients). According to the Baycan et al. study, PAI levels were higher in the non-survivor, survivor, and control groups, and they are associated with the hypo-fibrinolytic state observed in SARS-CoV-2 [34]. This impairment causes hypercoagulation and increases the risk of thrombosis, which suggests that PAI could be a key biomarker for predicting disease severity in COVID-19 patients.

The results of our investigation suggest that thrombomodulin is significantly higher in ICU patients than in non-ICU patients and asymptomatic patients with COVID-19 patients, indicating endothelium damage. Goshua et al. and Wool and Miller et al. found a similar pattern, highlighting that the increase in markers points towards a state of heightened endothelial injury and activation in ICU patients, which may play a role in the hypercoagulable state observed in severe cases of COVID-19 [35,36].

Our study has certain limitations that should be acknowledged. First, in this single-center trial, the number of participants in the three groups was relatively small, but the sample was more homogeneous. Furthermore, smoking status was not reported. Also, given the small sample size, the influence of age on disease severity was not assessed. In order to overcome these limitations and further validate our findings, we suggest that larger groups of patients be studied to confirm that P-selectin is a predictor and a stable factor of severity. Future studies should examine the association between P-selectin and other markers of endothelial permeability to improve the accuracy of disease severity prediction and classification.

In addition, our results are related to previous SARS-CoV-2 variants and are interesting to evaluate whether they remain robust to contemporary variants. P-selectin plays a multifaceted role and promotes microvascular thrombosis and angiogenesis caused by ischemic diseases such as cardiovascular, inflammatory bowel, and septic diseases.

Our study results underscore the importance of considering the broader implications of P-selectin in the context of thromboembolism, infection severity, and genetic variants. An important focus of research is the complex role of P-selectin in promoting the thrombosis and angiogenesis of microvascular diseases associated with vascular diseases, such as cardiovascular, intestinal, and septic diseases.

## 5. Conclusions

The COVID-19 pandemic has prompted extensive research on the prediction of disease severity, with numerous biomarkers under examination. Our investigation revealed that P-selectin consistently and reliably predicted disease severity among the various biomarkers examined in patients with distinct clinical characteristics of the Delta and Omicron variants. Elevated levels of P-selectin, D-dimer, FVIII, and PAI-1 were strongly associated with increased disease severity, particularly in ICU patients. These markers can predict the severity of the disease and improve early diagnosis and personalized treatment. Our research on endothelial dysfunction and angiogenesis markers, including P-selectin, broadly impacts various diseases affecting the endothelium, such as diabetes, arthritis, high blood pressure, and heart disease.

## Figures and Tables

**Table 1:** Demographic characteristics.

Group of Patients (n = 90 Patients)	Asymptomatic (n = 30 Patients)	Non-ICU (n = 30 Patients)	ICU (n = 30 Patients)
Age	27–75 years (mean: 43 years)		
Sex	51 males (56.7%) and 39 females (43.3%)		
Demographic characteristics	(Caucasians)		

**Table 2:** Mean–SD for standard laboratory tests in total number of patients (asymptomatic, non-ICU, ICU) (n = 90).

	Asymptomatic (n = 30)		Non-ICU (n = 30)		ICU (n = 30)		
	Mean	SD	Mean	SD	Mean	Mean	Units
HB	13.5	2.0	11.9	1.8	12.7	12.7	g/dL
HCT	40.7	6.0	36.2	5.2	30.2	6.5	%
WBC	6.4	2.7	10.2	6.3	17.2	12.1	K/μL
Neutrophils absolute count	4.1	2.3	8.5	5.9	14.5	11.8	K/μL
Lymphocytes absolute count	1.5	0.8	1.2	0.7	1.56	1.17	K/μL
Neutrophil/ Lymphocyte ratio (NLR)	3.19		10.44		15.86		0.78–3.53
PLT	222.3	62.7	243.9	132.0	263.5	263.5	K/μL

**Table 3:** Median, mean, and SD P-selectin values for the six subgroups of patients.

Subgroup	Median	Mean	SD
asymptomatic 2021	3.23	3.10	1.04
asymptomatic 2022	2.52	2.95	0.95
non-ICU 2021	5.52	5.96	1.57
non-ICU 2022	3.17	4.32	2.54
ICU 2021	10.47	11.4	6.40
ICU 2022	7.80	8.21	3.70

## Data Availability

The data presented in this study are available on request from the corresponding author. The data are not publicly available due to intellectual property concerns.

## Supplementary Materials

The following supporting information can be downloaded at: https://www.mdpi.com/article/10.3390/cimb47060409/s1.

## Abbreviations

Activated partial thromboplastin time (aPTT), Blood Dendritic Cell Antigen-3 (BDCA-3), deep venous thrombosis (DVT), enzyme-linked immunosorbent assay (ELISA), variants of concern (VOCs), plasminogen activator inhibitor-1 (PAI), plasminogen activator (tPA), prothrombin time (PT), real-time reverse transcription PCR (RT-PCR), soluble P-selectin (sCD62P), urokinase-type plasminogen activator (uPA), variance inflation factor (VIF), von Willebrand antigen (VWF: Ag), von Willebrand factor (VWF), von Willebrand factor ristocetin cofactor (VWF: RCo), World Health Organization (WHO), variants of interest (VOIs).
