# Supplementary material for: Biomarkers of Endothelial Damage and Disease Severity in COVID-19 Patients
Source: Curr Issues Mol Biol. 2025 May 31;47(6):409. doi: 10.3390/cimb47060409 (PMC12191880; doi:10.3390/cimb47060409)
Supplement: Supplementary file 1 [file cimb-47-00409-s001.zip › cimb-3610673-supplementary.pdf]

Supplementary Material

Table S1 History of infection

Group of patients (N=90 patients)	asymptomatic (n=30 patients)		non-ICU (n=30 patients)		ICU (n=30 patients)	
	2021	2022	2021	2022	2021	2022
History of infection	first	14 first<br>1 second at collection of samples	first	first	first	first

Table S2 Patients-Comorbidities

	outpatient (30 patients)	non –ICU (30 patients)	ICU (30 patients)
CVD	-	11 (36.6%)	10 (33.3%)
COPD	2 (6.6%)	1 (3.3%)	8 (26.6%)
Hematologic diseases	-	2 (6.6%)	1 (3.3%)
DM	-	-	3 (10%)
neurological disorders	-	3 (10%)	1 (3.3%)
autoimmune diseases	-	1 (3.3%)	1 (3.3%)
ARDS	-	6 (20%)	15 (50%)
Death	-	9 (4 CVD / 4 ARDS/1 neurological disorder)	28 (15 ARDS / 6 CVD/ 7 COPD)

CVD: Cardiovascular diseases (Hypertension - heart disease), COPD: Chronic obstructive pulmonary disease, DM: Diabetes mellitus, ARDS: Acute respiratory distress syndrome

Table S3 Patients and vaccination

Vaccination Status	Outpatients 2021	Outpatients 2022	Non-ICU 2021	Non-ICU 2022	ICU 2021	ICU 2022	Total
Unvaccinated	15 (100.0%)	7 (46.7%)	8 (53.3%)	3 (20.0%)	12 (80.0%)	6 (40.0%)	51 (56.7%)
1 dose	0 (0.0%)	0 (0.0%)	5 (33.3%)	2 (13.3%)	1 (6.7%)	2 (13.3%)	10 (11.1%)
2 doses	0 (0.0%)	1 (6.7%)	2 (13.3%)	0 (0.0%)	2 (13.3%)	1 (6.7%)	6 (6.7%)
3 doses	0 (0.0%)	7 (46.7%)	0 (0.0%)	10 (66.7%)	0 (0.0%)	6 (40.0%)	23 (25.6%)
Total (n)	15	15	15	15	15	15	90

Table S4. Median values for each patient group for PT, INR, aPTT, Fibrinogen, D-dimer.

	outpatients	non-ICU	ICU	Units
PT				
median	12.2	12.2	14.1	sec
INR				
median	1.04	1.04	1.20	
aPTT				
median	30.2	31.9	34.3	sec
Fibrinogen				
median	400	370	411	mg/dl
D-dimer				
median	179	399	838	ng/ml

Table S5. Median values for PAI, thrombomodulin and P-selectin.

	outpatients	non-ICU	ICU	Units
FVIII				
median	110.5	207.5	269	%
VWF: Ag				
median	190.5	209.5	361	

VWF: RCo				
median	200	235	306	
PAI				
median	6.82	6.58	8.31	ng/ml
thrombomodulin				
median	1646	1782	2347	pg/ml
P-selectin				
median	2.715	4.702	8.353	ng/ml

Table S6. Collinearity Diagnostics: Estimated Variance Inflation Factor (VIF) for the twelve indices, with the dependent variable being the severity of the patient.

Index	VIF	Index	VIF
PAI	1.22	fibrinogen	1.43
Thrombomodoulin	1.20	D-dimer	1.44
p-selectin	1.32	FVIII	1.54
PT	36.41	vWAg	38.19
INR	37.34	vWRecof	23.68
aPTT	1.51	VWF	9.913

Table S7. Parameter Estimates from ordinal logistic regression with independent variables: P-selectin, D-dimer, FVIII, and PAI, and dependent variable the severity of the patient.

							95% Confidence Interval	
		Estimate	Std. Error	Wald	df	Sig.	Lower Bound	Upper Bound
Threshold	[group = 1]	8.56	1.67	26.16	1	<.001	5.285	11.850
	[group = 2]	12.85	2.34	29.96	1	<.001	8.250	17.452
Location	P-selectin	0.62	0.14	17.85	1	<.001	0.335	0.914
	D-dimer	0.002	0.001	11.89	1	<.001	0.001	0.003
	FVIII	0.017	0.005	14.21	1	<.001	0.008	0.026
	PAI	0.343	0.110	9.70	1	0.002	0.127	0.559

Link function: Logit.
